# The Microbiota-Derived Metabolite of Quercetin, 3,4-Dihydroxyphenylacetic Acid Prevents Malignant Transformation and Mitochondrial Dysfunction Induced by Hemin in Colon Cancer and Normal Colon Epithelia Cell Lines

**DOI:** 10.3390/molecules25184138

**Published:** 2020-09-10

**Authors:** Mabel Catalán, Jorge Ferreira, Catalina Carrasco-Pozo

**Affiliations:** 1 Programa de Farmacología Moleculary Clínica, Instituto de Ciencias Biomédicas, Facultad de Medicina, Universidad de Chile, Santiago 7500000, Chile; mabelcatalan@u.uchile.cl (M.C.); jferreir@med.uchile.cl (J.F.); 2 Discovery Biology, Griffith Institute for Drug Discovery, Griffith University, Nathan 4111, Queensland, Australia

**Keywords:** 3,4-dihydroxyphenylacetic acid, hemin, quercetin, sulforaphane, colorectal cancer, malignant transformation and mitochondrial dysfunction

## Abstract

Meat diet plays a pivotal role in colorectal cancer (CRC). Hemin, a metabolite of myoglobin, produced after meat intake, has been involved in CRC initiation. The compound, 3,4-dihydroxyphenylacetic acid (3,4HPAA) is a scarcely studied microbiota-derived metabolite of the flavonoid quercetin (QUE), which exert antioxidant properties. The aim of this study was to determine the protective effect of 3,4HPAA against malignant transformation (increased cell proliferation, decreased apoptosis, DNA oxidative damage and augmented reactive oxidative species (ROS) levels) and mitochondrial dysfunction induced by hemin in normal colon epithelial cells and colon cancer cells. The effect of 3,4HPAA was assessed in comparison to its precursor, QUE and to a known CRC protective agent, sulforaphane (SFN). The results showed that both, tumor and normal cells, exposed to hemin, presented increased cell proliferation, decreased caspase 3 activity and cytochrome c release, as well as augmented production of intracellular and mitochondrial ROS. In addition, hemin decreased the mitochondrial membrane potential (MMP) and the activity of complexes I and II of the electron transport chain. These effects of hemin were prevented by the action of 3,4HPAA. The metabolite showed to be more active than QUE and slightly less active than SFN. In conclusion, 3,4HPAA administration could represent a promising strategy for preventing malignant transformation and mitochondrial dysfunction in colon epithelia induced by hemin.

## 1. Introduction

CRC is the third most commonly occurring cancer in men and the second most commonly occurring cancer in women [1]. The incidence has increased by 2% in the population aged <50 years. In fact, It has been estimated that the incidence rates for colon and rectal cancers will increase by 90.0%, and 124.2%, respectively by 2030 in people aged between 20 to 34 years [2]. In 2015, the Agency for Research on Cancer (IARC), an affiliated organization of the World Health Organization classified red meat as “probably carcinogenic to humans” and processed meat as “carcinogenic to humans”. The daily intake of 100 g of red meat increased the risk of CRC by 17% [3]. The excessive intake of heme iron should be considered, as it is an intrinsic factor of carcinogenicity, exhibited by dietary red meat. Hemin, a metabolite of heme produced in the gut after meat intake, has been involved in CRC initiation, as it promotes colon carcinogenesis through mechanisms involving oxidative damage [4,5,6,7].

There is a strong association with diets containing abundant vegetables, fruits, and grains, and a significant decline in the risk of colon cancer. The flavonol QUE (and QUE-glycosylated) is one of the most abundant polyphenols present in fruits and vegetables [8]. The anticancer effect of QUE in CRC is related to mechanisms involving cell cycle arrest, apoptosis, antioxidant response and regulation of signaling pathways [9,10]. QUE is barely absorbable in the gastrointestinal tract, and as a result, it accumulates in the colon lumen [11]. Colonic microbiota can degrade flavonoids, generating a large number of metabolites that can be absorbed and exert biological effects in the body, such as antioxidant, antimicrobial and anti-inflammatory activities, among others [12]. The major microbial metabolite of QUE and its glycosylated derivatives is 3,4-dyhydroxyphenylacetic acid (3,4HPAA) [13,14,15,16,17,18,19,20], which also has potent antioxidant properties. Although 3,4HPAA is abundantly produced in the colon, its role in CRC prevention remains unknown.

The isothiocyanate SFN is an isothiocyanate created from glucoraphanin (abundant in broccoli) by the action of myrosinase enzymes during crushing of the plant. Microbiota also has myrosinase activity, indicating that SFN can be formed from its precursor glucoraphanin in the colon [21]. SFN has extensively been shown to possess anticancer properties and to exert protection against CRC development in in vitro and in vivo models [22,23].

The present study aimed to determine the effect of 3,4HPAA against malignant transformation (increased cell proliferation, DNA oxidative damage and augmented reactive oxidative species (ROS) levels) and mitochondrial dysfunction induced by hemin in normal colon epithelial cells and colon cancer cells. We postulate that the beneficial effects of 3,4HPAA are associated with its antioxidant property and its ability to protect the mitochondrial function. This study also contributes to explaining the “health-promoting” effects of QUE, particularly against the development of CRC, and taking into account the metabolism that this flavonoid undergoes in the colon.

## 2. Results

### 2.1. SFN, QUE and 3,4HPAA Reduced, and Hemin Increased RKO and CCD841 Cell Viability

**Figure 1:**
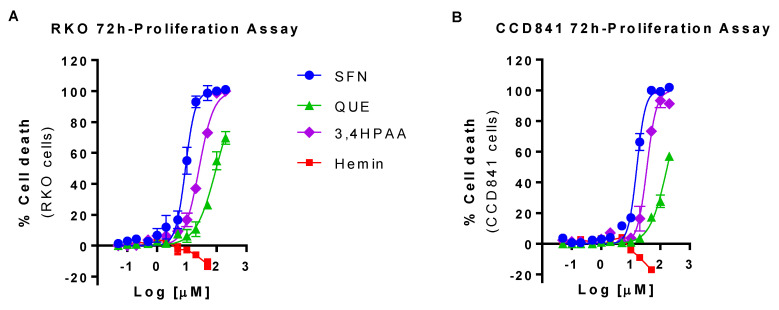
Effect of hemin, SFN, QUE and 3,4HPAA in cell viability. (**A**) RKO cells and (**B**) CCD841 cells were incubated with increasing concentrations of hemin (0.05–50 μM), SFN, QUE or 3,4HPAA (0.05–200 μM). After 72 h, the 3-(4,5-dimethylthiazol-2-yl)-5-(3-carboxymethoxyphenyl)-2-(4-sulfophenyl)-2H-tetrazolium) reduction was detected by absorbance (MTS assay). The results were expressed as percentage of inhibition in relation to the positive control (30 µM puromycin) and calculated as: ((OD_0.4% DMSO_ − OD_sample_) × 100)/(OD_0.4% DMSO_ − OD_puromycin_). Values are expressed as mean ± SEM, from three independent culture preparations. 3,4HPAA, 3,4-dihydroxyphenylacetic acid; QUE, quercetin; SFN, sulforaphane.

SFN, QUE and 3,4HPAA more efficiently reduced the cell viability in RKO than in CCD841 cells. In comparison to RKO cells, 20 and 37% higher concentration of SFN, and 3,4HPAA, respectively, was required to reduce by 50% the viability of CCD841 cells (SFN = IC_50 RKO cells_ 8.8 ± 0.21 μM and IC_50 CCD841 cells_ 16.12 ± 0.90 μM, *p* = 0.0014 t-test; 3,4HPAA = IC_50 RKO cells_ 25.8 ± 0.687 μM and IC_50 CCD841 cells_ 35.4 ± 3.33 μM, *p* = 0.0460 t-test) (Figure 1A,B). QUE IC_50_ values were only estimated as it didn’t reach the 100% of inhibition and it didn’t plateau at the tested concentrations. Based on this estimation, 72% higher concentration of QUE is needed to reduce the cell viability of CCD841 cells by 50%, in comparison to the RKO cells (IC_50 RKO cells_ 99.8 ± 13.1 μM and IC_50 CCD841 cells_ 172 ± 13.3 μM, *p* = 0.018, t-test) (Figure 1A,B). Hemin at 50 μM increased the cell viability of RKO and CCD841 by 12 and 17% respectively (RKO: *p* = 0.0139; CCD841 cells: *p* < 0.0001) (Figure 1A,B), which was prevented by SFN, QUE and 3,4HPAA (Figure S1).

In order to determine the effect of the compounds in apoptosis, oxidative stress and mitochondrial dysfunction under conditions in which their effect on cell proliferation is marginal, the compounds were further studied at the concentrations that caused 10% of inhibition (IC_10_) of RKO cells proliferation (SFN = 1 μM; QUE = 10 μM and 3,4HPAA = 2.6 μM). The IC_10_ values were determined (interpolated using Prism) from the 72-hr proliferation assay with RKO cells, Figure 1A). Hemin was further evaluated at 10 μM, maximal concentration that did not increase cell proliferation either in RKO cell or CCD841 cells after 72 h incubation (Figure 1A,B).

### 2.2. SFN, QUE and 3,4HPAA Prevented the Decrease of Apoptosis Induced by Hemin

**Figure 2:**
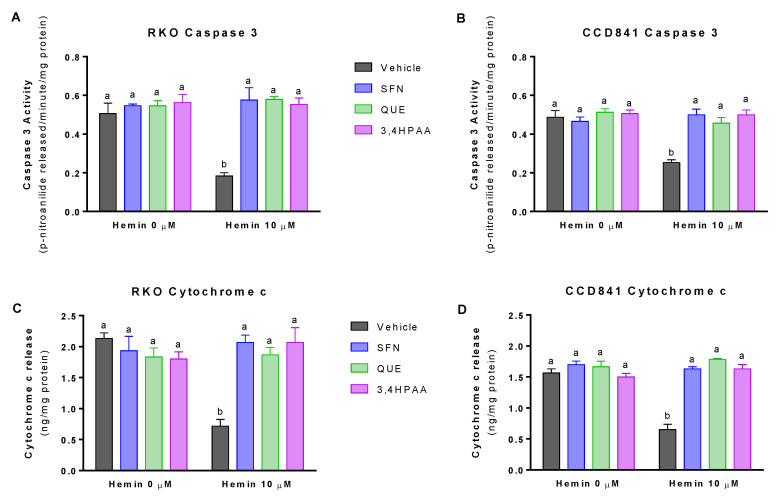
Effect of hemin, SFN, QUE and 3,4HPAA in apoptosis. Cells were incubated with vehicle (0.4% DMSO), 1 μM SFN, 10 μM QUE or 2.6 μM 3,4HPAA in the absence or presence of 10 μM hemin. After 72 h, caspase 3 activity was measured in (**A**) RKO cells and (**B**) CCD841 cells; as well as cytochrome c levels in the media of (**C**) RKO cells; and (**D**) CCD841 cells. Caspase activity and cytochrome c release were expressed as *p*-nitroanilide/min/mg of protein, and ng (cytochrome c)/mg of protein, respectively. Values are expressed as mean ± SEM, from three independent culture preparations. For all bars with the same letter, the difference between the means is not statistically significant. Values with different letters indicate significant differences (*p* < 0.05) between bars (two-way ANOVA, Bonferroni post-test). 3,4HPAA, 3,4-dihydroxyphenylacetic acid; QUE, quercetin; SFN, sulforaphane.

At 10 μM, hemin decreased caspase 3 activity by 68% in RKO cells and 48% in CCD841 cells (Figure 2A,B). Hemin (10 μM) also decreased cytochrome c release by 66% in RKO cells and 59% in CCD841 cells (Figure 2C,D). SFN, QUE and 3,4HPAA, at the concentrations of 1, 10 and 2.6 μM, respectively prevented the decrease of caspase 3 and cytochrome c induced by hemin, in both cell lines (Figure 2A–D). Unlike 3,4HPAA, QUE at a concentration of 2,6 μM (Figure S2) did not prevent the decrease in apoptosis induced by hemin. In the same line, 3,4HPAA at 1 μM failed to protect antiapoptotic changes promoted by hemin (data not shown).

### 2.3. SFN, QUE and 3,4HPAA Inhibited the Hemin-Induced Increase in ROS Levels and DNA/RNA Oxidation

**Figure 3:**
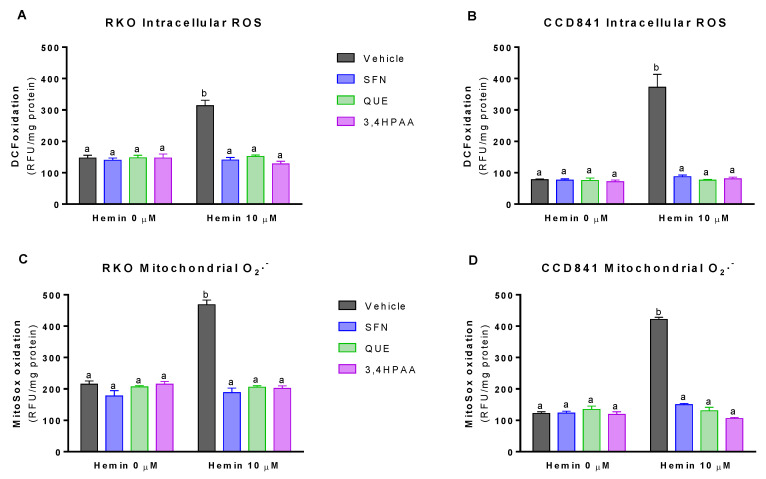
Effect of hemin, SFN, QUE and 3,4HPAA in ROS levels. Cells were incubated with vehicle (0.4% DMSO), 1 μM SFN, 10 μM QUE or 2.6 μM 3,4HPAA in the absence or presence of 10 μM hemin. After 72 h, DCF oxidation was measured by fluorescence in (**A**) RKO cells; and (**B**) CCD841 cells; as well as MitoSoxTM Red oxidation in (**C**) RKO cells; and (**D**) CCD841 cells. The results were expressed as RFU/mg of protein. Values are expressed as mean ± SEM, from three independent culture preparations. For all bars with the same letter, the difference between the means is not statistically significant. Values with different letters indicate significant differences (*p* < 0.05) between bars (two-way ANOVA, Bonferroni post-test). 3,4HPAA, 3,4-dihydroxyphenylacetic acid; DCF, dichlorofluorescein; QUE, quercetin; SFN, sulforaphane; RFU, relative fluorescence unit.

Basal (vehicle in the absence of hemin) intracellular ROS levels and mitochondrial O_2_^−^ levels were 76 and 88% greater in RKO cells than in CCD841 cells, respectively (*p* = 0.0016 t-test, Figure 3A,B; *p* = 0.0013 t-test, Figure 3C,D). At 10 μM, hemin increased intracellular ROS levels by 113% in RKO cells and 383% in CCD841 cells (Figure 3A,B), which were completely inhibited by 1 μM SFN, 10 μM QUE and 2.6 μM 3,4HPAA. Same situation was observed for the mitochondrial O_2_^−^ levels, in which hemin increased this free radical by 118 and 245% in mitochondria from RKO and CCD841 cells, respectively (Figure 3C,D).

**Figure 4:**
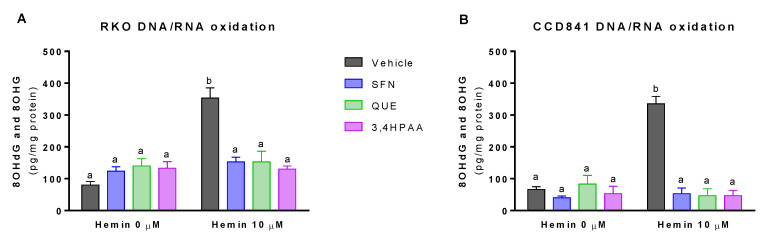
Effect of hemin, SFN, QUE and 3,4HPAA in DNA/RNA damage. Cells were incubated with vehicle (0.4% DMSO), 1 μM SFN, 10 μM QUE or 2.6 μM 3,4HPAA in the absence or presence of 10 μM hemin. After 72 h, 8OHdG and 8OHG levels were assessed in (**A**) RKO; and (**B**) CCD841 cell supernatants. The results were expressed as pg (8OHdG and 8OHG)/mg of protein. Values are expressed as mean ± SEM, from three independent culture preparations. For all bars with the same letter, the difference between the means is not statistically significant. Values with different letters indicate significant differences (*p* < 0.05) between bars (two-way ANOVA, Bonferroni post-test). 3,4HPAA, 3,4-dihydroxyphenylacetic acid; 8OHdG, 8-hydroxy-2′-deoxyguanosine; 8OHG, 8-hydroxyguanosine; QUE, quercetin; SFN, sulforaphane.

Basal levels of 8OHdG and 8OHG were 20% greater in RKO than in CCD841 cell supernatant (*p* = 0.0277 t-test, Figure 4A,B). Hemin, at a concentration of 10 μM, increased DNA/RNA oxidation by 4.4-fold and 5-fold in RKO cells and CCD841 cells, respectively (Figure 4A,B). This effect of hemin was fully inhibited by 1 μM SFN, 10 μM QUE and 2.6 μM 3,4HPAA in both cell lines (Figure 4A,B).

SFN, QUE and 3,4HPAA had no effect on the ROS or 8OHdG and 8OHG levels in the absence of hemin (Figure 3A–D and Figure 4A,B). Unlike 3,4HPAA, QUE tested at 2.6 μM didn’t protect against the ROS levels and DNA/RNA oxidation induced by hemin in RKO or CCD841 cells (Figure S3). Same lack of effect was found with 1 μM 3,4HPAA (data not shown).

### 2.4. SFN, QUE and 3,4HPAA Inhibited the Hemin-Induced Decrease in MMP and Complex I and Complex II Activities

**Figure 5:**
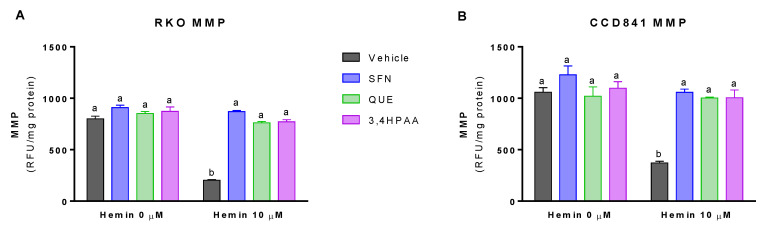
Effect of hemin, SFN, QUE and 3,4HPAA in MMP. Cells were incubated with vehicle (0.4% DMSO), 1 μM SFN, 10 μM QUE or 2.6 μM 3,4HPAA in the absence or presence of 10 μM hemin. After 72 h, MMP was measured by fluorescence in (**A**) RKO; and (**B**) CCD841 cell supernatants. Results were expressed as RFU/mg of protein. Values are expressed as mean ± SEM, from three independent culture preparations. For all bars with the same letter, the difference between the means is not statistically significant. The values with different letters indicate significant differences (*p* < 0.05) between bars (two-way ANOVA, Bonferroni post-test3,4HPAA, 3,4-dihydroxyphenylacetic acid; MMP, mitochondrial membrane potential; QUE, quercetin; SFN, sulforaphane; RFU, relative fluorescence unit.

**Figure 6:**
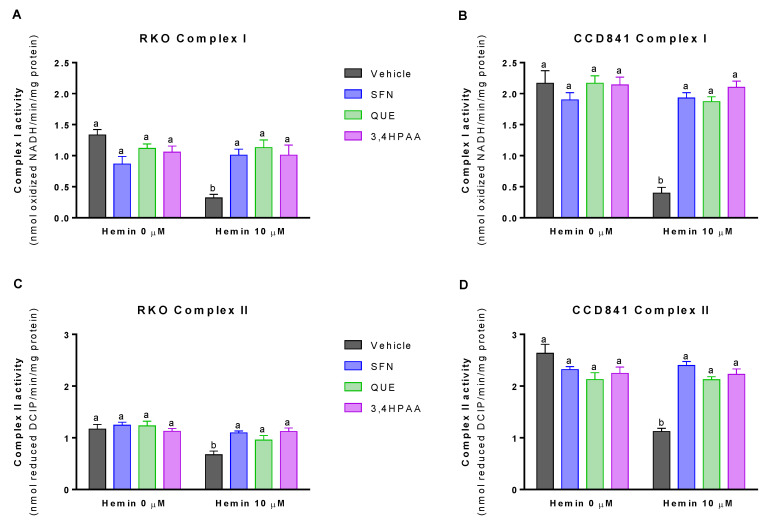
Effect of hemin, SFN, QUE and 3,4HPAA in complex I and II activities. Cells were incubated for 72 h with vehicle (0.4% DMSO), 1 μM SFN, 10 μM QUE or 2.6 μM 3,4HPAA in the absence or presence of 10 μM hemin. Complex I activity was measured in (**A**) RKO cells; and (**B**) CCD841 cells and results were expressed as nmol of oxidized NADH/minute/mg of protein. Complex II activity was measured in (**C**) RKO cells; and (**D**) CCD841 cells and results were expressed as nmol of reduced DCIP/minute/mg of protein. Values are expressed as mean ± SEM, from three independent culture preparations. For all bars with the same letter, the difference between the means is not statistically significant. Values with different letters indicate significant differences (*p* < 0.05) between bars (two-way ANOVA, Bonferroni post-test). 3,4HPAA, 3,4-dihydroxyphenylacetic acid; DCIP, 2,6-dichlorophenolin-dophenol; QUE, quercetin; SFN, sulforaphane.

Basal (vehicle in the absence of hemin) levels of MMP was 32% greater in CCD841 than in RKO cells (*p* = 0.0072 t-test, Figure 5A,B). In addition, basal activities of the complex I and II were 63 and 125% higher in CCD841 than in RKO cells (Complex I: *p* = 0.0196 t-test, Figure 6A,B; Complex II: *p* = 0.018 t-test, Figure 6C,D). Hemin reduced MMP (Figure 5A) and the activities of complex I (Figure 6A) and complex II (Figure 6C) by 74, 76, and 43% in RKO cells, respectively; whilst in CCD841 cells, hemin reduced by 65% MMP (Figure 5B), 82% complex I activity (Figure 6B) and 57% complex II activity (Figure 6D). These effects of hemin were fully inhibited by 1 μM SFN, 10 μM QUE and 2.6 μM 3,4HPAA (Figure 5A,B and Figure 6A–D).

SFN, QUE and 3,4HPAA had no effect on the cells in the absence of hemin (Figure 5A,B and Figure 6A–D). Unlike 3,4HPAA, QUE at a concentration of 2.6 μM (Figure S4) didn’t protect against the reduction of MMP and complex activities induced by hemin in RKO or CCD841 cells. 3,4HPAA at 1 μM did’t prevent these mitochondrial alterations promoted by hemin (data not shown)

## 3. Discussion

CRC is the third most commonly occurring cancer in men and the second most commonly occurring cancer in women [1]. Interestingly, more than 50% of all CRC cases and deaths are attributable to modifiable risk factors, such as unhealthy nutritional lifestyles [4]. In terms of bowel cancers, in 2015, the World Health Organization classified processed meats as a Group 1 carcinogen (known to cause cancer); while red meat has been classified as a Group 2A carcinogen (probable to cause cancer). Hemin, a metabolite of heme produced in the gut after meat intake, has been involved in CRC initiation, as it promotes colon carcinogenesis through mechanisms involving oxidative damage [4,5,6,7]. It has been proposed that iron present in heme or hemin is a key promotor of CRC through its ability to initiate lipid peroxidation and to produce nitroso compounds [24]. In the present study, we postulate that 3,4HPAA, a microbiota-derived metabolite of QUE, may protect colon cells in vitro, against malignant transformation and mitochondrial dysfunction induced by hemin. This protective effect could be associated with the antioxidant property of this metabolite and its ability to prevent mitochondrial dysfunction.

### 3.1. 3,4HPAA Prevented the Hemin-Induced Increase Proliferation of Normal Colon Epithelial Cells and Colon Cancer Cells

It has been widely demonstrated that heme induces colon epithelial hyperproliferation and decreases apoptosis [25,26,27]. Also it has been proposed that heme induces compensatory hyperproliferation by damaging surface epithelial cells and the signals to crypt cells to promote aberrant growth [25,26]. Interestingly, Sesink et al. showed that dietary heme enhances cytotoxicity in rat colons through the involvement of a heme-induced cytotoxic metabolite [7]. This metabolite, identified as hemin, is a covalently modified porphyrin formed from heme in the gastrointestinal tract [6,7]. Therefore, it has been suggested that heme iron, in the form of hemin may explain the link between the risk of CRC and red meat intake, and the lack of a link with white meat intake [5,7]. Hemin supplementation to non-initiated rats increases fat peroxidation and cytotoxic activity of fecal water, and epithelial proliferation by 70% [7]. Therefore, in terms of evaluating the role of meat diet in CRC development, studying hemin in in vitro models is more physiologically relevant than studying heme. Consistently, we found that hemin increases the proliferation of normal colon epithelia and colon cancer cells (Figure 1), by reducing apoptosis (Figure 2) and that it was prevented in the presence of 3,4HPAA.

Our results showed that 3,4HPAA reduces the proliferation of colon cancer RKO cells after 72 h incubation (Figure 1), which is consistent with previous studies. While, 3,4HPAA has been shown to exhibit a potent antiproliferative effect on colon cancer HCT116 cells (IC_50_ 90 µM) after 24 h of incubation [28]. Skrbe et al. showed that the growth of HT29 colon cells decreased by 60% after 72 h of exposure to 100 µM 3,4HPAA [29]. Our results showed that 3,4HPAA reduces proliferation of colon cancer RKO cells more efficiently than of normal colon epithelia CCD841 cells (Figure 1). The clinical response of DNA-targeted chemotherapy is positively correlated to the proliferation rate of the tumour cells [30]. Although, more studies are needed to determine the mechanism of action underlying the anti-proliferative effect of 3,4HPAA, its effect could be DNA targeted, thus affecting cell division, as doubling time of RKO is 24 h and CCD841 is 72 h. Consistently, the anti-proliferative effect of 3,4HPAA is evidenced after allowing enough time for cell replication, as this effect is not observed in shorter incubation times. 3,4HPAA incubated for 6 h at 200 or 250 µM had no effect in the viability of mouse hepatoma Hepa1c1c7 cells [31] and Min-6 pancreatic β-cells [32], respectively. In addition, when incubated for only 2 h, 3,4HPAA (up to 1 mM concentration) did not affect the viability of Caco-2 cells [33].

### 3.2. 3,4HPAA Prevented the Intracellular and Mitochondrial ROS Increase and DNA Oxidative Damage Induced by Hemin in Normal Colon Epithelial Cells and Colon Cancer Cells

Our results showed that hemin increased ROS production in the cell and mitochondria of colon epithelial cells and colon cancer cells (Figure 3) [34,35,36,37]. Consistent with our finding, Gonzalez-Reyes et al. reported that 30 µM hemin increases ROS production in primary cultures of cerebellar granule neurons of rats [38]. This effect of hemin was completely abolished by the presence of the antioxidant molecules, curcumin [38] or 3,4HPAA (as we shown in Figure 3). 3,4HPAA has been shown to have the highest free radical scavenging activity among several flavonoid metabolites tested in vitro [39]. In addition, we have previously described that 3,4HPAA can enhance the cellular antioxidant defense, by increasing Nrf2 translocation to the nucleus in pancreatic *β*-cells [32]. Nrf2 regulates the component transcriptions of the glutathione and thioredoxin antioxidant systems, as well as the phase I and phase II detoxification systems [40]. Although, more studies are needed addressing the mechanism underlying the antioxidant effect of 3,4HPAA in this model, it is suggested that 3,4HPAA could reduce ROS levels induced by hemin through direct (ROS scavenging effect) and indirect antioxidant effects (Nrf2 antioxidant system).

Hemin-produced free iron reacts with hydrogen peroxide, through the Fenton reaction, to generate highly oxidizing species capable of initiating the oxidation of macromolecules, such as lipids, proteins and DNA/RNA [41]. In fact, we found that hemin induced DNA/RNA oxidation in colon epithelial cells and colon cancer cells (Figure 4). Consistently, it has been found that 25 µM hemin induces lipid peroxidation and protein oxidation in bovine aortic endothelial cells (BAEC), only after 4 h incubation [42]. This effect of hemin in lipid and protein oxidation was totally inhibited by the antioxidant α-tocopherol [42] In line with the protective effect exerted by antioxidants against oxidative stress induced by hemin, we found that 3,4HPAA fully prevented the DNA/RNA oxidation induced by hemin in colon epithelial cells and colon cancer cells (Figure 4).

In general, tumour cells exhibit higher levels of ROS and oxidative stress in comparison to normal cells [43], and the level of oxidative stress is directly correlated to the frequency of p53 mutation in CRC [44]. In our study, we found that colon cancer RKO cells, which is wild-type p53 [45,46], show similar ROS and oxidative stress levels than the normal CCD841 colon epithelia cells (Figure 3). Consistent with our finding, RKO cells have shown to exert the same ROS levels than the normal human umbilical vein endothelial cells (HUVEC) [47].

### 3.3. 3,4HPAA Prevented Mitochondrial Dysfunction Induced by Hemin in Normal Colon Epithelial Cells and Colon Cancer Cells

Our results showed that hemin caused mitochondrial dysfunction, by decreasing MMP (Figure 4) and complex I and II activities in normal colon epithelial cells and colon cancer cells (Figure 6). Hemin (50 µM for 1 h) has also been shown to reduce MMP in BAEC, which was associated to a decrease in the basal and maximal respiration [42]. This effect of hemin in mitochondrial respiration could be due to its ability to decrease complex I and II activities, as we reported here for the first time (Figure 6).

We showed that hemin induces the production of O_2_^−^ in mitochondria, a fact consistent with the alteration of mitochondrial function induced by this molecule (Figure 3). The idea that hemin increases mitochondrial O_2_^−^ production by inhibiting complex I and II is supported by the fact that rotenone and malonate (complex I, and II inhibitors, respectively) also promote the mitochondrial production of O_2_^−^ [48,49,50]. Mitochondrial O_2_^−^ is generated by the interaction between oxygen and reducing equivalents [51]. When the electron flow through the electron transport chain slows down, the proton efflux through the inner mitochondrial membrane diminishes (hemin decreased MMP), causing an increase of electron donors in mitochondria. In consequence, the concentration of oxygen increases (hemin decreased oxygen consumption [42]) and the formation of O_2_^−^ by the respiratory chain also increases (Figure 3C,D).

We have shown that mitochondrial dysfunction induced by hemin is prevented by 3,4HPAA (Figure 5 and Figure 6). Notably, 3,4HPAA may protect against hemin-induced ROS increase (Figure 3A,B), oxidative damage (Figure 4) and mitochondrial dysfunction (Figure 5 and Figure 6) by preventing O_2_^−^ production within mitochondria (Figure 3C,D), and protecting the activities of complex I and II (Figure 6).

### 3.4. Physiological Relevance of This Study and Limitations

The effect of 3,4HPAA reported here could be exerted in vivo, as the concentration, used in this study (2.6 μM), could be reached in the colon, considering that 6.98 µM of this metabolite has been detected in fecal water after the administration of a standard diet [52]. It was shown that 3,4HPAA is four times more effective than QUE in preventing the damage induced by hemin (Figures S2–S4). Therefore, it is possible that the metabolite is the main contributor to the protective effect against CRC observed with QUE in in vivo models [9,10]. SFN has extensively been shown to possess anticancer properties and to exert protection against CRC development in in vitro and in vivo models [22,23]. In our study, 3,4HPAA was shown to be only 2.6-fold less active than SFN, suggesting that the metabolite is of relevance for CRC protection and that the co-administration of SFN and QUE is highly beneficial to reduce CRC risk. However, further studies in vivo models are required to support the beneficial role of 3,4HPAA in CRC protection.

## 4. Materials and Methods

### 4.1. Materials

3,4-dihydroxyphenylacetic acid, quercetin, sulforaphane hemin, rhodamine123 (rhod), dichlorofluorescein (DCF) diacetate, triphenyltin,1,1,3,3-Tetramethoxypropane, 2,6-dichlorophenolin-dophenol (DCIP), rotenone, NADH, succinate, malonate and puromycin were purchased from Sigma-Aldrich (St. Louis, MO, USA). CellTiter96^®^ AQueous Assay (Madison, WI, USA) was obtained from Promega (Madison, Wi, USA) and DNA/RNA oxidation ELISA kit from Cayman Chemicals (Ann Arbour, MI, USA). Pierce BCA Protein Assay Kit was purchased from Thermo Fisher Scientific (Waltham, MA, USA). MitoSox^TM^ Red mitochondrial was obtained from Molecular Probes (Eugene, OR, USA).

### 4.2. Cell Culture and Treatments

Cells were cultured in MEM supplemented with 10% FBS, 100 IU/mL penicillin, 100 μg/mL streptomycin in a humidified atmosphere of 95% air, and 5% CO_2_. Cells were obtained from ATCC. RKO cells were seeded in a 96-well plate at a density of 1 × 10^4^ cells/well and the CCD841 at 4 × 10^4^ cells/well. After 24 h, cells were incubated for 24 h, 48 h and 72 h with SFN, QUE and 3,4HPAA in the absence or presence of hemin. However, compounds incubated for 24 h and 48 h did not cause alterations in the parameters measured. Only the results of 72 h incubation are shown. Reagents were diluted in DMSO, giving a final concentration in the cell plate of 0.4% DMSO (vehicle).

### 4.3. Cell Viability

Cell viability was measured through the reduction of MTS to a colored formazan dye that is soluble in media. Cells were treated with increasing concentrations of SFN, QUE and 3,4HPAA (0.05–200 μM). Maximum concentration of hemin tested was 50 μM, due to solubility limitation and colour interference with the assay readouts. After washing, the ability of cells to reduce MTS was evaluated by measuring the optic density (OD) at λ 490 nm, using the CellTiter96^®^ AQueous Assay according to the manufacturer’s instructions. The percentage of inhibition was calculated in relation to the positive control (30 µM puromycin) as: ((OD_0.4% DMSO_ − OD_sample_) × 100)/(OD_0.4% DMSO_ − OD_puromycin_). OD values were normalized to the negative and positive control. Dose-response curves (log(inhibitor) versus response, four parameters variable slope) were obtained using Graphpad Prism 7.0 (GraphPad Inc., La Jolla, CA, USA).

### 4.4. Apoptosis

Apoptosis was quantitated by measuring the activities of caspase- 3 (Millipore Corporation, Bedford, MA, USA) and cytochrome c. The catalytic activity of caspase-3 was measured independently by using a commercial colorimetric assay according to the manufacturer’s instructions [53]. The determination is based on the detection of the chromophore *p*-nitroanilide at 405 nm after its enzymatic cleavage from the labeled substrate DEVD-*p*-nitroanilide. The specific caspase- 3 inhibitor,0.1 mM Ac-DEVD-CHO, were used as enzymatic activity controls, according to the manufacturer’s instructions. Enzyme activity was calculated as µmol of *p*-nitroanilide released/minute/mg of protein. Cytochrome c was measured using a Cytochromec Kit ELISA assay (Life Technologies, CA, USA), according to manufacturer’s instructions [53].

### 4.5. Cellular Redox Status

The redox status of RKO and CCD841 cells were evaluated by measuring the intracellular reactive oxygen species and the cytosolic and mitochondrial O_2_^−^ production. The intracellular ROS was measured through the conversion of DCF into a fluorescent dye (λEx_485 nm_ and λEm_530 nm_) [32,54]. Mitochondrial O_2_^−^ production was evaluated through the oxidation of MitoSox^TM^ Red, a cationic lipophilic fluorochrome (λEx_510 nm_ and λEm_570 nm_) which accumulates in the mitochondrial matrix [32]. After treatments, cells were incubated at 37 °C for 30 min with 50 mM DCFD or 5 μM MitoSox™. Cells were washed and the fluorescence was measured using a Multi-Mode Microplate Reader (SynergyHT,BioTek) and data were normalized to the protein content. In order to evidence the specificity of MitoSox^TM^ for O_2_^−^, a control incubating the samples with SOD 100 U/mL for 10 min was also carried out (data not shown) [55]. In the presence of SOD, the MitoSox^TM^ oxidation was only 10–15%, indicating that under this experimental condition the oxidation of this probe induced by H_2_O_2_ was marginal. The H_2_O_2_-independent MitoSox^TM^ oxidation was calculated as URF_MitoSoxTM oxidation_ -URF_MitoSoxTM oxidation in the presence of SOD_.

### 4.6. DNA and RNA Oxidation

The levels of 8-hydroxy-2′-deoxyguanosine (8OHdG) and 8-hydroxyguanosine (8OHG) in cell media were assessed using DNA and RNA oxidation ELISA kit according to the manufacturer’s instructions. Values were normalized to the protein content using the Pierce BCA Protein Assay Kit.

### 4.7. Mitochondrial Membrane Potential

MMP was evaluated using Rhod, a cationic lipophilic fluorochrome (λEx_500 nm_ and λEm_530 nm_) which accumulates in the mitochondrial matrix. After treatments, cells were incubated with 1 mg/mL Rhod for 10 min at 37 °C. The specific accumulation of Rhod in the mitochondria was evaluated using as control 3 mM triphenyltin, an inductor of mitochondrial membrane permeability transition pores [32]. The fluorescence was measured using a Multi-Mode Microplate Reader (Synergy HT, BioTek). Protein content was determined by using the Pierce BCA Protein Assay Kit.

### 4.8. Complexes I and II Activities

Activities of complexes I and II of the electron transport chain were measured in mitochondria isolated from RKO and CCD841 cells as previously described [32,49,56]. Briefly, cells were harvested, washed in a Ca^2+^/Mg^2+^-free PBS and centrifuged (10 min; 1000 g; 4 °C). The pellet was resuspended and homogenized in a buffer solution (pH 7.4) containing 250 mM sucrose, 1 mM EGTA, 10 mM HEPES and 1mg/mL BSA (fraction V). The homogenate was centrifuged (10 min; 12,000 g; 4 °C); the supernatant discarded; and the pellet was washed and centrifuged as above. Complex I activity was measured in isolated mitochondria by determining the changes of OD_340 nm_ due to NADH oxidation (*ε* = 6.81 mM^−1^ cm^−1^). Rotenone (a complex I inhibitor) was used as a control; and the enzyme activity as rotenone-sensitive NADH–ubiquinone oxidoreductase was calculated. The activity of complex II was quantified by the increase in DCIP reduction measured at 600 nm (*ε* = 19.1 mM^−1^ cm^−1^); malonate (a complex II inhibitor, final concentration of 20 mM) was used as a control; and the enzyme activity as malonate-sensitive succinate-coenzyme Q reductase was calculated.

### 4.9. Statistical Analysis

Two-way ANOVA were performed followed by Bonferroni’s Multiple Comparison Test. The experiments were performed using three independent culture preparations; and in quadruplicate for RKO cell evaluations and duplicate for CCD841 cell assays. Values are expressed as mean ± SEM. Values with different superscript letters (a, b and c) indicate significant differences (*p* < 0.05) between groups. For all bars with the same letter, the difference between the means is not statistically significant.

## 5. Conclusions

In conclusion, this study shows that hemin induces cell proliferation, oxidative damage and mitochondrial dysfunction, as well as inhibits apoptosis in colon cancer and normal colon epithelia cell lines. 3,4HPAA, a microbial metabolite of QUE, due to its antioxidant and mitochondrial protective effects, prevented hemin-induced malignant transformation and mitochondrial impairment. Colonic metabolism of QUE seems to be critical for the CRC preventive effect of the flavonoid. We consider that 3,4HPAA administration could represent a promising strategy for preventing malignant transformation and mitochondrial dysfunction in colon epithelia induced by hemin. However, additional experiments are required to evaluate 3,4HPAA protective effects in in vivo models of CRC induced by meat diet.

## Supplementary Materials

The following are available online, Figure S1: Effect of SFN, QUE and 3,4HPAA in cell viability, in the presence of hemin, Figure S2: Comparison of the effect of QUE and 3,4HPAA in apoptosis, Figure S3: Comparison of the effect of QUE and 3,4HPAA in ROS levels, Figure S4: Comparison of the effect of QUE and 3,4HPAA in mitochondrial parameters.
