# Supplementary material for: The Microbiota-Derived Metabolite of Quercetin, 3,4-Dihydroxyphenylacetic Acid Prevents Malignant Transformation and Mitochondrial Dysfunction Induced by Hemin in Colon Cancer and Normal Colon Epithelia Cell Lines
Source: Molecules. 2020 Sep 10;25(18):4138. doi: 10.3390/molecules25184138 (PMC7571211; doi:10.3390/molecules25184138)
Supplement: Supplementary file 1 [file molecules-25-04138-s001.pdf]

The Microbiota-Derived Metabolite of Quercetin, 3,4-Dihydroxyphenylacetic Acid Prevents Malignant Transformation and Mitochondrial Dysfunction Induced by Hemin in Colon Cancer and Normal Colon Epithelia Cell Lines

Mabel Catalán [1], Jorge Ferreira [1] and Catalina Carrasco-Pozo [2,*]

[1] Programa de Farmacología Moleculary Clínica, Instituto de Ciencias Biomédicas, Facultad de Medicina, Universidad de Chile, 7500000 Santiago, Chile; mabelcatalan@u.uchile.cl (M.C.); jferreir@med.uchile.cl (J.F.)

[2] Discovery Biology, Griffith Institute for Drug Discovery, Griffith University, Nathan 4111, Queensland, Australia

* Correspondence: c.carrascopozo@griffith.edu.au; Tel.: +61-7-373-56034

Field Code Changed

Field Code Changed

Field Code Changed

Academic editors: Silvie Rimpelová; Riccardo Petrelli
Received: 5 August 2020; Accepted: 4 September 2020; Published: date

SUPPLEMENTARY FIGURE 1

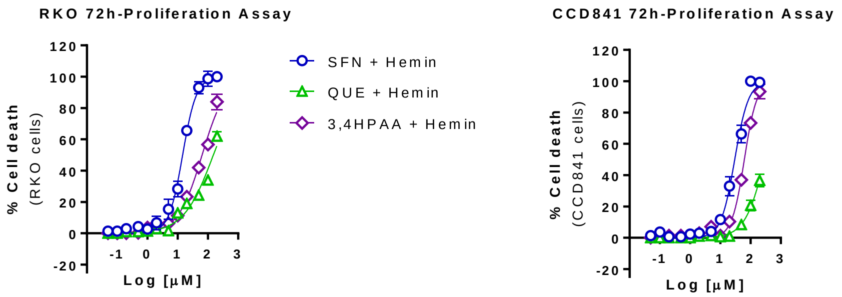

Figure S1. Effect of SFN, QUE and 3,4HPAA in cell viability, in the presence of hemin. (A) RKO cells and (B) CCD841 cells were incubated with increasing concentrations of SFN, QUE or 3,4 HPAA (0.05 – 200 μM), in the presence of 10 μM hemin. After 72h, the MTS reduction was detected by absorbance. The results were expressed as percentage of inhibition in relation to the positive control (30 μM puromycin) and calculated as: $((OD_{0.4\%\ DMSO} - OD_{sample})^{*} \times 100)/(OD_{0.4\%\ DMSO} - OD_{puromycin})$. Values are expressed as mean ± SEM, from three independent culture preparations. 3,4HPAA, 3,4-dihydroxyphenylacetic acid; QUE, quercetin; SFN, sulforaphane.

SUPPLEMENTARY FIGURE 2

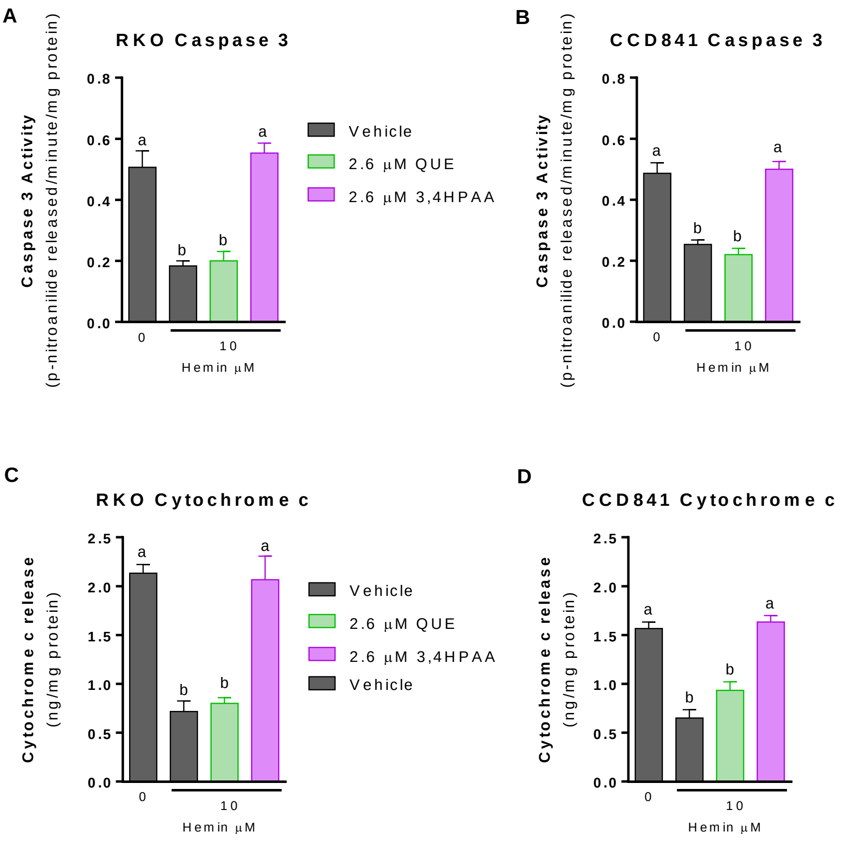

Figure S2. Comparison of the effect ~~Effect~~ of QUE and 3,4HPAA in apoptosis. Cells were incubated with vehicle (0.4% DMSO), 10 µM hemin, 2.6 µM QUE + 10 µM hemin or 2.6 µM 3,4HPAA + 10 µM hemin. After 72h, caspase 3 activity was measured in (A) RKO cells and (B) CCD841 cells; as well as cytochrome c levels in the media of (C) RKO cells and (D) CCD841 cells. Caspase activity and cytochrome c release were expressed as p-nitroanilide/minute/mg of protein and ng (cytochrome c)/mg of protein, respectively. Values are expressed as mean ± SEM, from three independent culture preparations. For all bars with the same letter, the difference between the means is not statistically significant. Values with different letters indicate significant differences ($p < 0.05$) between bars (two-way ANOVA, Bonferroni post-test). 3,4HPAA, 3,4-dihydroxyphenylacetic acid; QUE, quercetin.

SUPPLEMENTARY FIGURE 3

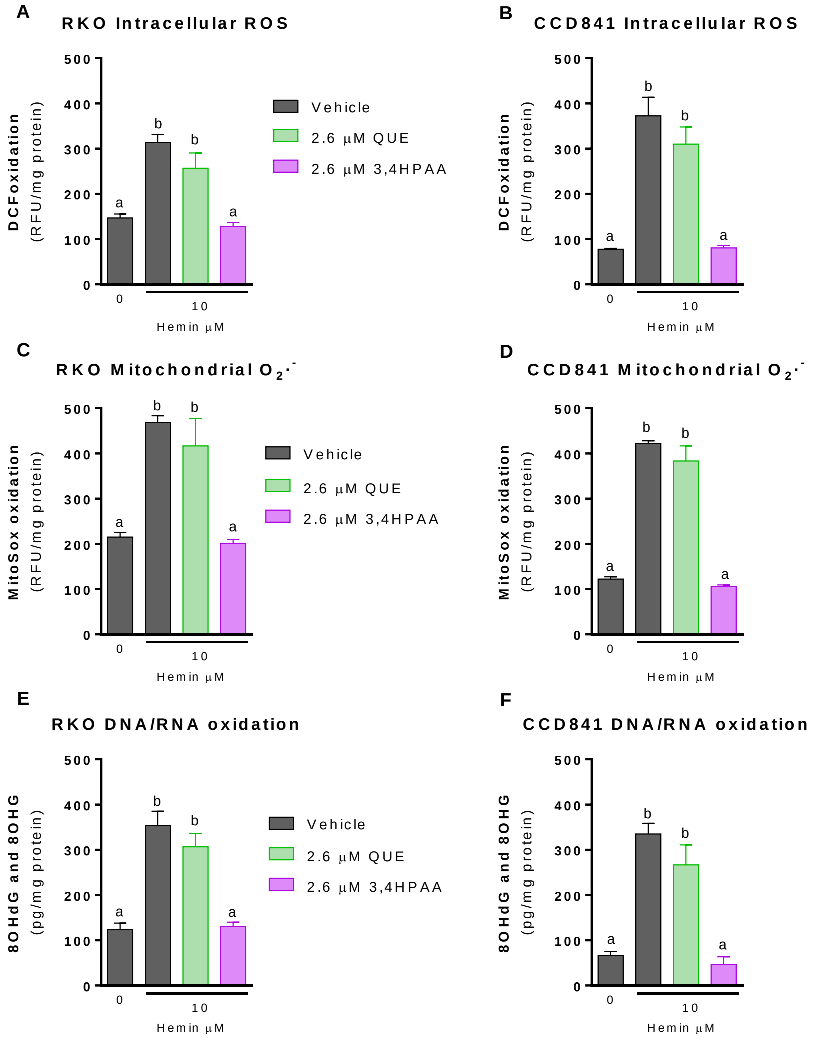

Figure S3. Comparison of the effect ~~Effect~~ of QUE and 3,4HPAA in ROS levels. Cells were incubated with vehicle (0.4% DMSO), 10 µM hemin, 2.6 µM QUE + 10 µM hemin or 2.6 µM 3,4HPAA + 10 µM hemin. After 72h, DCF oxidation was measured by fluorescence in (A) RKO cells and (B) CCD841 cells; as well as MitoSoxTM Red oxidation in (C) RKO cells and (D) CCD841 cells. 8OHdG and 8OHG levels were assessed in (E) RKO and (F) CCD841 cell supernatants. DCF and MitoSox oxidations were expressed as RFU/mg of protein; DNA/RNA oxidation was expressed as pg (8OHdG and 8OHG)/mg of protein. Values are expressed as mean ± SEM, from three independent culture preparations. For

all bars with the same letter, the difference between the means is not statistically significant. Values with different letters indicate significant differences ($p < 0.05$) between bars (one-way ANOVA, Bonferroni post-test). 3,4HPAA, 3,4-dihydroxyphenylacetic acid; 8OHdG, 8-hydroxy- 2'-deoxyguanosine; 8OHG, 8-hydroxyguanosine; DCF, dichlorofluorescein; QUE, quercetin; RFU, relative fluorescence unit.

SUPPLEMENTARY FIGURE 4

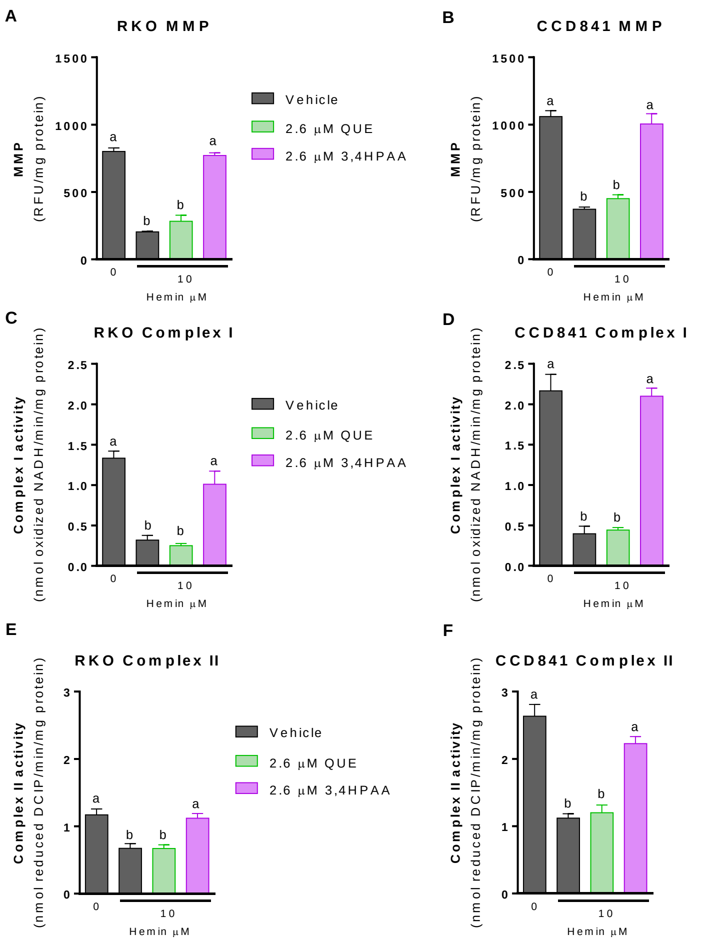

Figure S4. Comparison of the effect ~~Effect~~ of QUE and 3,4HPAA in mitochondrial parameters. Cells were incubated with vehicle (0.4% DMSO), 10 μM hemin, 2.6 μM QUE + 10 μM hemin or 2.6 μM 3,4HPAA + 10 μM hemin. After 72h, MMP was measured by fluorescence in (A) RKO cells and (B)

CCD841 cells and expressed as RFU/mg of protein. Complex I activity was measured in (C) RKO cells and (D) CCD841 cells and results were expressed as nmol of oxidized NADH/minute/mg of protein. Complex II activity was measured in (E) RKO cells and (F) CCD841 cells and results were expressed as nmol of reduced DCIP/minute/mg of protein. Values are expressed as mean ± SEM, from three independent culture preparations. For all bars with the same letter, the difference between the means is not statistically significant. Values with different letters indicate significant differences ($p < 0.05$) between bars (one-way ANOVA, Bonferroni post-test). 3,4HPAA, 3,4-dihydroxyphenylacetic acid; DCIP, 2,6-dichlorophenolin-dophenol; MMP, mitochondrial membrane potential; QUE, quercetin; RFU, relative fluorescence unit.
